# Detection of Mild Cognitive Impairment Using Convolutional Neural Network: Temporal-Feature Maps of Functional Near-Infrared Spectroscopy

**DOI:** 10.3389/fnagi.2020.00141

**Published:** 2020-05-21

**Authors:** Dalin Yang, Ruisen Huang, So-Hyeon Yoo, Myung-Jun Shin, Jin A. Yoon, Yong-Il Shin, Keum-Shik Hong

**Affiliations:** ^1^School of Mechanical Engineering, Pusan National University, Busan, South Korea; ^2^Department of Rehabilitation Medicine, Pusan National University School of Medicine and Biomedical Research Institute, Pusan National University Hospital, Busan, South Korea; ^3^Department of Rehabilitation Medicine, Pusan National University School of Medicine, Pusan National University Yangsan Hospital, Yangsan-si, South Korea

**Keywords:** functional near-infrared spectroscopy (fNIRS), mild cognitive impairment (MCI), convolutional neural network (CNN), temporal feature, brain map, *N*-back, Stroop, verbal fluency task

## Abstract

Mild cognitive impairment (MCI) is the clinical precursor of Alzheimer's disease (AD), which is considered the most common neurodegenerative disease in the elderly. Some MCI patients tend to remain stable over time and do not evolve to AD. It is essential to diagnose MCI in its early stages and provide timely treatment to the patient. In this study, we propose a neuroimaging approach to identify MCI using a deep learning method and functional near-infrared spectroscopy (fNIRS). For this purpose, fifteen MCI subjects and nine healthy controls (HCs) were asked to perform three mental tasks: *N*-back, Stroop, and verbal fluency (VF) tasks. Besides examining the oxygenated hemoglobin changes (ΔHbO) in the region of interest, ΔHbO maps at 13 specific time points (i.e., 5, 10, 15, 20, 25, 30, 35, 40, 45, 50, 55, 60, and 65 s) during the tasks and seven temporal feature maps (i.e., two types of mean, three types of slope, kurtosis, and skewness) in the prefrontal cortex were investigated. A four-layer convolutional neural network (CNN) was applied to identify the subjects into either MCI or HC, individually, after training the CNN model with ΔHbO maps and temporal feature maps above. Finally, we used the 5-fold cross-validation approach to evaluate the performance of the CNN. The results of temporal feature maps exhibited high classification accuracies: The average accuracies for the *N*-back task, Stroop task, and VFT, respectively, were 89.46, 87.80, and 90.37%. Notably, the highest accuracy of 98.61% was achieved from the ΔHbO slope map during 20–60 s interval of *N*-back tasks. Our results indicate that the fNIRS imaging approach based on temporal feature maps is a promising diagnostic method for early detection of MCI and can be used as a tool for clinical doctors to identify MCI from their patients.

## Introduction

Alzheimer's disease (AD) is authoritatively listed as the sixth leading cause of death in the United States (US), and it is also the fifth primary cause of death for those aged 65 years and above (Taylor et al., 2017). Seven hundred thousand people aged 65 years and above in the US were estimated death based on AD in 2019 (Hebert et al., 2013). As recently reported by Alzheimer Association, it estimated 18.5 billion hours of assistance (valued at $233.9 billion) was provided by the caregivers of people with AD or other dementias (Alzheimer Association, 2019). It is thought that AD starts at least 20 years before the symptoms occur with small unnoticeable changes in the brain. Symptoms arise because of the damaged nerve cells (neurons) related to thinking, learning, and memory (Gordon et al., 2018). Symptoms tend to grow over time and gradually start to interfere with the ability of an individual to perform everyday activities until death. AD is considered a progressive, irreversible neurological brain disorder. Currently, no pharmacological treatment exists that can decelerate or prevent the symptoms of AD (Alzheimer Association, 2019). Many researchers suppose that the early stage in the AD process, at either the mild cognitive impairment (MCI) or preclinical stage, will be the most effective period for future treatments to slow down or prevent the progression of AD (Yiannopoulou and Papageorgiou, 2013). Thus, it is essential to assess biomarkers (i.e., the indication of the medical state observed from outside of patients; Strimbu and Tavel, 2010) for identifying individuals who are in these early stages of the disease and can receive appropriate treatment.

There are three categories of diagnostic biomarkers for AD, which are named β-amyloid-Aβ deposits (A), hyperphosphorylated tau aggregates (T), and neurodegeneration or neuronal injury (N) (Jack et al., 2018). The ATN synopsis is widely assessed through cerebrospinal fluid (CSF) or medical imaging. Thus far, no evidence that supports the preeminence of any biomarker over another (CSF vs. imaging) for the diagnostic assessment of AD exists. The selection of biomarkers typically relies on the cost, availability, and convenience of tests (Khoury and Ghossoub, 2019). However, because medical imaging can identify the different stages of the AD temporally and anatomically, some researchers claim that the superiority of medical imaging over the biofluid biomarkers mentioned above (Márquez and Yassa, 2019).

Functional near-infrared spectroscopy (fNIRS) is a non-invasive neuroimaging technique, which is used to measure activation-induced changes in the cerebral hemoglobin concentrations of oxyhemoglobin (ΔHbO) and deoxyhemoglobin (ΔHbR) (Perrey, 2014; Shin and Im, 2018; Hong et al., 2020). The blood flow and oxygen metabolism are induced by the neural activity in the neighboring capillary network (Hong et al., 2014; Zafar and Hong, 2018; Ghafoor et al., 2019). In comparison with the existing neuroimaging techniques involving direct neural activation measurement methods such as magnetoencephalography (MEG) and electroencephalography (EEG) (Kumar et al., 2019), fNIRS offers the advantage of higher spatial resolution and lower susceptibility to the movement artifact (Naseer and Hong, 2015; Wilcox and Biondi, 2015; Hong et al., 2018; Pfeifer et al., 2018). In contrast, other well-established neuroimaging techniques are typically associated with the metabolism of biochemical components during neural activity and exist a limitation in terms of temporal resolution. These techniques include positron emission tomography (PET), single-positron emission computed tomography (SPECT), and functional magnetic resonance imaging (fMRI) (Strangman et al., 2002). In particular, because of the property requirement of the radioactive isotopes, PET and SPECT do not allow continuous or repeated measurements, a factor that also limits their application in the cases of children and pregnant women (Irani et al., 2007). Although fMRI is non-radiative and involves no risk, it is physically constraining, is sensitive to movement artifacts, exposes participants to an excessively noisy environment, and is expensive (Ferrari and Quaresima, 2012). These features render fMRI inappropriate for certain research and many clinical applications (Santosa et al., 2014). In contrast, fNIRS is a novel neuroimaging modality with the following advantages: it is non-invasive, safe, less costly, portable, and tolerant of motion artifacts (Perrey, 2008); it also has great temporal resolution and moderate spatial resolution (Ghafoor et al., 2017; Zafar and Hong, 2020). In addition, fNIRS is in progress to improve the spatial and temporal resolutions with the development of bundled-optodes configurations (Nguyen and Hong, 2016; Nguyen et al., 2016), detection of the initial dip (Zafar and Hong, 2017; Hong and Zafar, 2018), and combination of adaptive method (Iqbal et al., 2018; Hong and Pham, 2019; Pamosoaji et al., 2019) to improve information transfer rate.

In the past decades, the fNIRS study of psychiatric or neural-disorder patients highly depended on mass-univariate analytical techniques such as statistical parametric mapping (Vieira et al., 2017). Traditionally, the research studies compared the hemodynamic response of a patient with that of healthy control (HC) and determined neuroanatomical or neurofunctional differences at the group level. Most AD/MCI detection studies typically employed the ΔHbO/ΔHbR (Jahani et al., 2017; Perpetuini et al., 2017; Vermeij et al., 2017; Katzorke et al., 2018; Yoon et al., 2019) and relative temporal features such as the mean value, slope value, number of active channels, peak location, skewness, and kurtosis (Yap et al., 2017; Li et al., 2018a), and they determined the significant differences for comparison. The straightforwardness and interpretability of this methodology has led to considerable advances in our comprehension of the neurological disorders. With the development of technology, the following limitations of mass-univariate analytical techniques have been revealed. (1) Statistical information is extracted according to each region of interest (ROI) channel based on the assumption that various brain regions perform independently. In practice, this assumption is inconsistent with brain function (Biswal et al., 2010). The network-level comparison explains the neurological symptoms better than the focal-level comparison (Mulders et al., 2015). (2) Statistical analysis cannot easily yield individual diagnosis results (Vieira et al., 2017). Mass-univariate techniques are suitable only for detecting differences between groups. According to the evaluation conducted in our initial study, the results of statistical analysis are not consistent with the classification results (Yang et al., 2019). Thus, an effective classification method based on fNIRS neuroimaging is crucial for the detection of MCI in the clinical stage.

Deep learning (DL) has allowed significant progress in the identification and classification of image patterns and is considered a promising machine-learning methodology (Ravi et al., 2017). Convolutional neural networks (CNNs), the most broadly used DL architecture, have delivered excellent performances in computer-aided prediction for neurological disorders (Mamoshina et al., 2016; Tanveer et al., 2019). The great success of CNNs in neural-image classification and analysis, which evidences their strong image-classification ability (Cecotti and Gräser, 2011; Ieracitano et al., 2018; Lin et al., 2018; Waytowich et al., 2018; Oh et al., 2019), motivated us to develop a CNN-based classification method for early-stage AD detection. So far, there are not any discussions in the literature, which used the DL method as an assistive tool for the diagnosis of early-stage AD by fNIRS signals, except for our group.

In our initial investigation (Yang et al., 2019), we compared the hemodynamic responses and statistical information between the groups of MCI and HC: We evaluated the digital biomarkers (i.e., mean, slope, peak, kurtosis, and skewness) and image biomarkers (i.e., *t*-map and connectivity map) for MCI identification. The MCI group showed decreased ΔHbO responses in comparison with the HC group, which is consistent with the literature (Vermeij et al., 2017; Katzorke et al., 2018). As digital biomarkers, 15 features (i.e., mean value of ΔHbO for 5–65 s, mean value of ΔHbR for 5–65 s, mean value of ΔHbO for 5–25 s, mean value of ΔHbR for 5–25 s, mean value of ΔHbO for 0–peak time, slope of ΔHbO for 5–15 s, slope of ΔHbR for 5–15 s, slope of ΔHbO for 20–60 s, slope of ΔHbR for 20–60 s, slope of ΔHbO for 60–70 s, slope of ΔHbR for 60–70 s, slope of ΔHbO for 0–peak time, peak time itself, skewness of ΔHbO for 5–65 s, and kurtosis of ΔHbO for 5–65 s) were introduced for the statistical analysis and three brain regions (i.e., left, middle, and right prefrontal brain regions) were examined. Some of the features (e.g., mean value of ΔHbO for 5–65 s in the right prefrontal brain region with *N*-back task) indicated a significant difference (*p* < 0.05) between the MCI and HC groups. For classification, linear discriminant analysis (LDA) was used. The highest accuracy out of three mental tasks (i.e., *N*-back task, Stroop task, and VFT) was 76.67% from *N*-back and Stroop tasks, which were based on manually selected ROI channels. Also, we evaluated the *t*-map and connectivity map as image biomarkers. The CNN result based on *t*-maps of the *N*-back task achieved the best performance of 90.62%. Based upon these findings, the conclusion was that image biomarkers like *t*-map or connectivity map provide a better classification accuracy than digital biomarkers. Motivated on this, we will investigate whether the combined digital biomarkers on a given space (i.e., mean-value image in a specified time interval, or slope-value image in a specific time interval, etc.) can provide an improved classification accuracy than the *t*-map result obtained in the previous work.

In this study, we investigated 63 types of neural images based on temporal (3 types), spatial (39 types), and temporal-spatial (21 types) features of fNIRS signals, which were acquired based on three mental tasks—the *N*-back, Stroop, and verbal fluency tasks (VFT)—for the early detection of AD via a CNN. The temporal features refer to the raw ΔHbO in time series, neuroimaging in the spatial domain refers to the brain map generated at specific time points, and the temporal-spatial features represent temporal features (mean value, slope value, skewness, and kurtosis) in the spatial domain. To the best of the author's knowledge, this is the first fNIRS neuroimaging study integrating digital biomarkers in a spatial domain, in which the diagnosis performance for early AD detection via a DL approach has been explored.

## Methods

**Figure 1:**
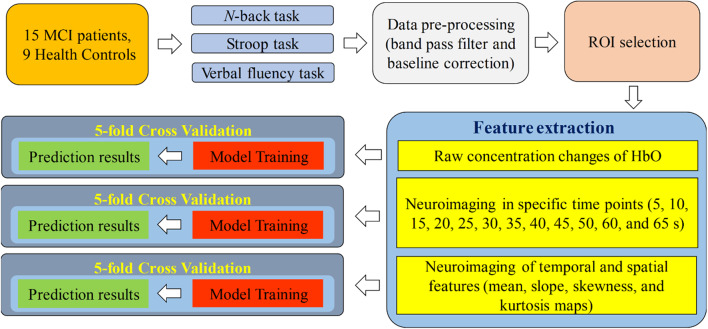
Systematic diagram of the proposed system.

Figure 1 presents a diagram of the proposed system. fNIRS data were acquired while the subjects were performing the three aforementioned mental tasks. After the signal preprocessing, the ROI channels were selected for the subsequent steps. In the feature-extraction step, the raw concentration changes in HbO, neuroimaging at a specific time point (i.e., 5, 10, 15, 20, 25, 30, 35, 40, 45, 50, 55, 60, and 65 s) in the spatial domain and neuroimaging of temporal features (mean value of ΔHbO for 5–65 s, mean value of ΔHbO for 5–25 s, slope of ΔHbO for 5–15 s, slope of ΔHbO for 20–60 s, slope of ΔHbO for 60–70 s, skewness of ΔHbO for 5–65 s, and kurtosis of ΔHbO for 5–65 s) in the spatial domain were generated for training the CNN model separately. Finally, 5-fold cross-validation was employed to assess the performance of the CNN model trained by the features mentioned above.

### Participants

In this study, 15 MCI patients (1 male and 14 females) and 9 HCs (2 males and 7 females) were recruited from the Pusan National University Hospital (Busan, South Korea). All 24 subjects are right-handed, able to communicate in Korean, similar ages, and educational backgrounds. The mental health state of each participant was assessed using three criteria: the Korean-mini-mental state examination (K-MMSE) (Han et al., 2008), the Seoul Neuropsychological Screening Battery (Ahn et al., 2010), and magnetic resonance imaging (MRI) data. Table 1 shows the summarized demographic information for 24 participants, comprising age (mean ± SD), gender, educational background (mean ± SD), statistical information, and K-MMSE scores (mean ± SD). The experiment was performed consistently with the approval of the Pusan National University Institutional Review Board (General Assembly of the World Medical Association, 2013). All the subjects were provided with a comprehensive explanation of the whole experimental contents before the start of the experiment. After the introduction, they were asked to write consent agreeing of the test.

**Table 1:** Demographic information of participants.

**Characteristics**	**MCI (*n* = 15)**	**HC (*n* = 9)**	***p*-value^a^**
Gender (Male/Female)	1/14	2/7	0.44
Education [years]	11.2 (±4.81)	10.56 (±2.88)	0.36
Age [years]	69.27 (±7.09)	68.33 (±4.69)	0.36
K-MMSE Score	25.13 (±2.33)	27.22 (±1.98)	0.49

*K-MMSE, Korea Mini-Mental State Examination*.

a *Two sample t-test with a significant level of 0.05*.

### Experimental Paradigm

**Figure 2:**
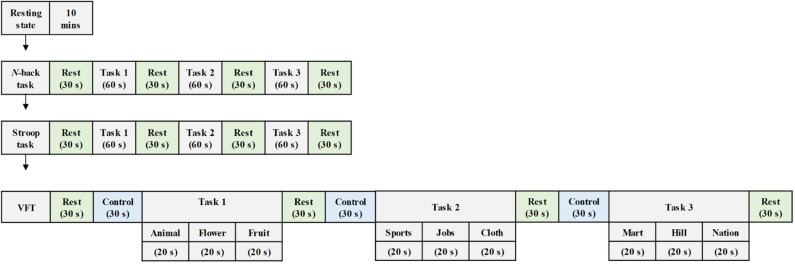
Experiment paradigm for various mental tasks (i.e., *N*-back task, Stroop task, and verbal fluency task) during the examination.

As shown in Figure 2, the experiment comprised three mental task sections, where each section consisted of three trials. In this study, the *N*-back task was used to assess working memory (Kane et al., 2007). The ability to inhibit cognition was evaluated by the Stroop task. This suppression occurs when the other attribute of the same stimulus simultaneously effects during the processing of a stimulus (McVay and Kane, 2009; Scarpina and Tagini, 2017). The performance of semantic verbal fluency task indicated the ability of the vocabulary size, lexical access speed, updating, and inhibition for each subject (Shao et al., 2014).

Participants were asked to sit on a comfortable chair and were directed to avoid movement. Each task trial took 60 s, and a 30 s rest was given between tasks. First, the subjects enjoyed a 10 m resting state before a task-based experiment section began. Then, they performed the 2-back version of the *N*-back task wherein a digital number between one and nine was randomly showed on the screen. When the current number matched the second to last number previously displayed on display, the participants were instructed to press the keyboard. The subjects were then asked to execute the Stroop task. The Korean-color word Stroop test (K-CWST) was utilized in this study. The participants were requested to read the color of letters within a limited time. Those letters were written by four different colors, i.e., red, blue, yellow, and black, respectively. Finally, the subjects executed the semantic VFT by generating as many words as possible within 1 min; the words should relate to the given semantic category. The amount of information of participants, which can be retrieved based on the categorization and memorial source of text during the limited time, were measured during this task.

### fNIRS Data Acquisition

**Figure 3:**
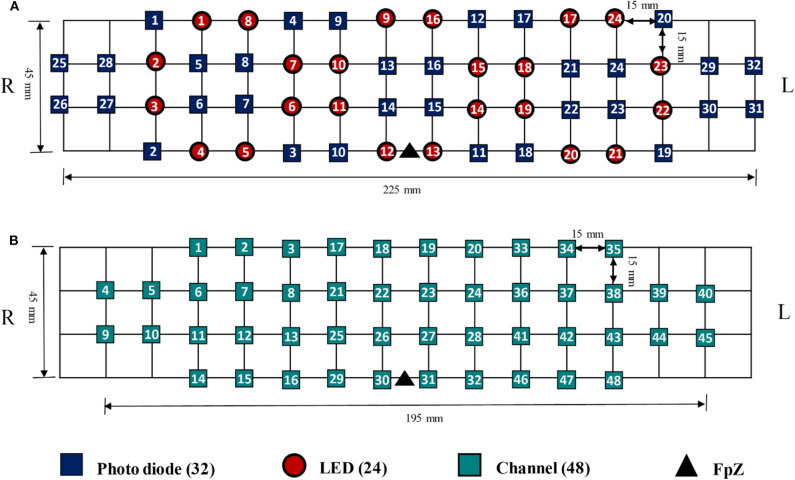
**(A)** The placement of Emitter and detector, **(B)** channel configuration with FpZ as a reference point based on the 10–20 international system.

The data utilized in this study were acquired by NIRSIT (OBELAB Inc., Rep. of Korea), which is a near-infrared multi-channel continuous wave system using a sampling rate of 8.138 Hz. The wavelengths employed for detecting two chromophores (i.e., oxygenated hemoglobin and deoxygenated hemoglobin) were 780 and 850 nm, respectively. A total of 24 emitters and 32 detectors, the placements of which are illustrated in Figure 3A, were used to measure the neural activation of the prefrontal cortex comprehensively. In total, 48 channels were selected for covering the entire prefrontal cortex. The channel configuration, illustrated in Figure 3B, was set up in accordance with the international 10–20 EEG system with the reference point FpZ. The pairs of emitter and detector (one channel) were placed 30 mm apart.

### fNIRS Data Pre-processing

The modified Beer-Lambert law was utilized to convert the optical densities to ΔHbO and ΔHbR (Sassaroli and Fantini, 2004). The converted signals passed 4th-order Butterworth low- and high-pass filters (i.e., cutoff frequencies: 0.001 and 0.1 Hz, respectively) to remove physiological noise, i.e., cardiac noise−1 Hz, respiration−0.25 Hz, and Mayer signal−0.1 Hz (Naseer et al., 2016; Khan and Hong, 2017; Liu et al., 2018; Nguyen et al., 2018). In accordance with our previously published evaluation results (Yang et al., 2019) and the relevant literature (Hoshi, 2007), it was observed that ΔHbO is more sensitive and dependable than ΔHbR. Besides, ΔHbO shows a stronger correlation with the fMRI BOLD response than ΔHbR (Cui et al., 2011; Li et al., 2018a). Therefore, ΔHbO signals were used because their signal-to-noise ratio was higher than that of ΔHbR signals.

As the limitation of the spatial resolution (as compared to fMRI), the ROIs—the areas that are active during the mental task—must be estimated. ROI analyses have been widely as a means of testing prior hypotheses above brain function in fMRI and PET areas; they enhance the statistical power as compared to entire brain area analyses and facilitate comparisons through multiple participants (Mitsis et al., 2008). In this study, the ROI was defined by the weighting factor (*t*-value) between the desired hemodynamic response function (dHRF) and the fNIRS measurement. The measurement (*y*) can be represented by the linear relationship of the dHRF with the coefficients and the error (ε), as shown in Equation (1). The dHRF was generated by convoluting the canonical hemodynamic response function (using two gamma functions) with the stimulation duration (i.e., the 60 s task and 30 s rest period). The *t*-value (*t*) was calculated using the *robustfit* function of MATLAB^TM^. The null hypothesis is β_1_ = 0, and SE represents standard error. ROI channels (i.e., activated channel) were selected when the calculated *t*-value was higher than the critical *t*-value (tcrt = 1.9632). The critical *t*-value was computed by the degree of freedom of the signals and statistical significance (*p* < 0.05 for two-sided tests).

(1) $$\begin{array}{l} y=\left[1\;dHRF\right]\left[\begin{array}{c} \beta_{0}\\ \beta_{1} \end{array}\right]+\varepsilon , \end{array}$$

(2) $$\begin{array}{l} t=\left[\frac{\beta_{0}}{SE\left(\beta \right)},\;\frac{\beta_{1}}{SE\left(\beta \right)}\;\right]. \end{array}$$

### Feature Extraction

**Figure 4:**
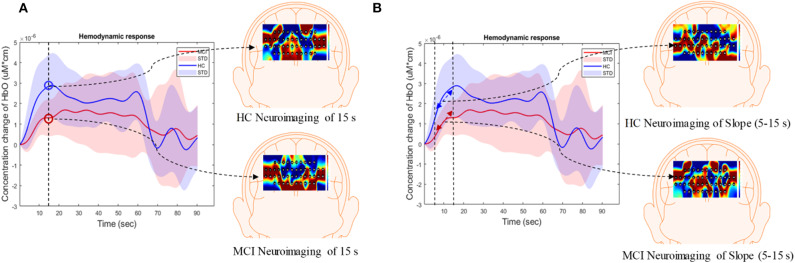
Overview of **(A)** spatial features and **(B)** temporal-spatial features using the hemodynamic response of ΔHbO.

In this study, the extracted features were divided into three categories: temporal, spatial, and temporal-spatial features. Temporal features referred to the raw ΔHbO in the time series and were considered to contain the concentration change in ΔHbO with time. The spatial feature describes neuroimaging at the specific time points. In this study, we selected 13 time points (i.e., 5, 10, 15, 20, 25, 30, 35, 40, 45, 50, 55, 60, and 65 s) to create the neural image for comparison purposes. The spatial feature indicates the neural activation at a specific time point in the spatial domain (prefrontal brain cortex). Figure 4A illustrates an example of neuroimaging at the 15 s time point. In this study, the selected time windows between 5 and 65 s were considered the effect of initial time delay (3–5 s) during the hemodynamic response.

**Figure 5:**
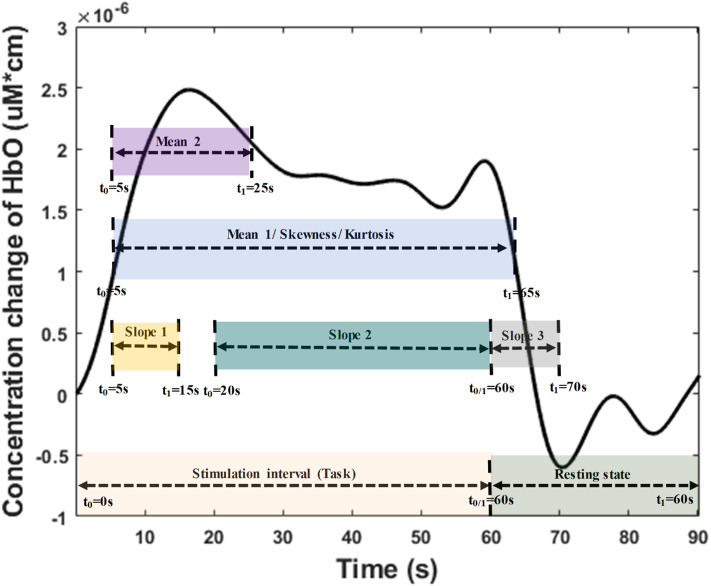
Time interval distribution of the temporal features (i.e., mean, slope, skewness, and kurtosis) for temporal-spatial neural images generation.

The temporal-spatial feature expresses the temporal information (mean value of ΔHbO for 5–65 s, mean value of ΔHbO for 5–25 s, slope of ΔHbO for 5–15 s, slope of ΔHbO for 20–60 s, slope of ΔHbO for 60–70 s, skewness of ΔHbO for 5–65 s, and kurtosis of ΔHbO for 5–65 s) in the spatial domain as shown in Figure 5. In other words, they display the values of specific time points according to the channel placement in the prefrontal cortex. Figure 4B illustrates the neuroimaging of the slope map for the period between 5 and 15 s. The mean value distinguishes the difference in the neural activation of the MCI and HC. Since the initial peak time of the hemodynamic response typically occurs during the time windows of the first 20 s, the time interval of 5–25 s was chosen. The slope features, i.e., the slope maps of 5–15 s, 20–60 s, and 60–70 s, were selected based on the characteristic from three intervals of the hemodynamic response: the initial increasing, plateau, and final period of ΔHbO, i.e., 5–15 s, 20–60 s, and 60–70 s, respectively. The slope indicates the difference in speed of activation between two groups, MCI and HC. Lastly, the difference in the asymmetry and the point of the probability distribution were measured by skewness (i.e., from 5 to 65 s) and kurtosis (i.e., from 5 to 65 s). These measurements are intended to investigate the overall difference of hemodynamic responses between MCI patients and HCs. All temporal information (mean, slope, skewness, and kurtosis) was determined by utilizing functions of *mean, polyfit, skewness*, and *kurtosis*, respectively, based on the MATLAB^TM^.

### Convolutional Neural Network

CNN is a special type of feedforward neural network. It takes advantage of local spatial coherence in the input, which allows the model to include fewer weights because of the parameter-sharing strategy (Cecotti and Gräser, 2011; Kim and Choi, 2019; Oh et al., 2019). In addition, CNN can learn features automatically from the input images by adjusting the parameters to minimize classification errors (Trakoolwilaiwan et al., 2017; Liu and Stathaki, 2018; Moon et al., 2018). Typically, CNN comprises convolutional, activation, pooling, and fully connected layers (Yi et al., 2018; Kim et al., 2019). Convolutional layers are the crucial component of CNN. Suppose the input is *X* with the 2-dimensional image (*h* × *w*), and the weight matrices (called kernels) have the size (*k*_1_ × *k*_2_), the local input region X_i_ can be converted to feature map (*Y*_j_) as shown in Equation (3), and size is *y*_1_ × *y*_2_.

(3) $$\begin{array}{l} Y_{j\;}=f(\sum X_{i}●K_{j}{+\;\beta }_{j}), \end{array}$$

where (●) denotes the convolution operator, and β_*j*_ is the bias term. One feature map (*Y*_*j*_) would be generated based on the sharing parameters of the *j*-th kernel with stride *s*. Thus, the size of the feature map can be calculated by using:

(4) $$\begin{array}{l} y_{1\;}=\;\frac{h-k_{1}+2\;\times p\;}{s}+1\;, \end{array}$$

(5) $$\begin{array}{l} y_{2\;}=\;\frac{w-k_{2}+2\;\times p\;}{s}+1\;, \end{array}$$

where *p* refers to the parameter of zero padding. This parameter is applied to keep the size of the output and input the same by padding the input edges with zeros. The activation layers are utilized after the convolutional layer. Typically, a non-linear transfer function called rectified linear units (*ReLu*) is widely used to achieve a better performance in regard to generalization and learning time (Yarotsky, 2017; Ieracitano et al., 2018). The function is shown in Equation (6). Thus, the feature map transfers the negative activation to be zero.

(6) $$\begin{array}{l} f\left(x\right)=\;max\left(o,\;x\right)\;. \end{array}$$

There are two options in the pooling layer—average pooling and maximum pooling—that are used to reduce the resolution of the input feature map. As discussed in the literature (Sun et al., 2017), the effectiveness of maximum pooling is significantly superior to average pooling because of the ability to capture invariant features and better generalization performance. For this reason, we also employed maximum pooling in this study. The output (*z*_1_ × *z*_2_) of the pooling layer is as follows:

(7) $$\begin{array}{l} z_{1\;}=\;\frac{y_{1}-\;p_{1}}{s_{p}}+1, \end{array}$$

(8) $$\begin{array}{l} z_{2\;}=\;\frac{y_{2}-\;p_{2}\;}{s_{p}}+1, \end{array}$$

where *s*_p_ is the stride of maximum pooling, and *p*_1_ = *p*_2_ represents the pooling size. Drop-out is applied for improving the CNN performance and avoiding overfitting. In this layer, the input and output are the same size. It is randomly initialized to turn the on or off of the corresponding neuron of the CNN at the beginning of the training iteration. As in the standard DL method, each neuron of a fully connected layer is connected with the previous layer. Since this is the issue of two group classification, there are two neurons for the last fully connected layer.

**Figure 6:**
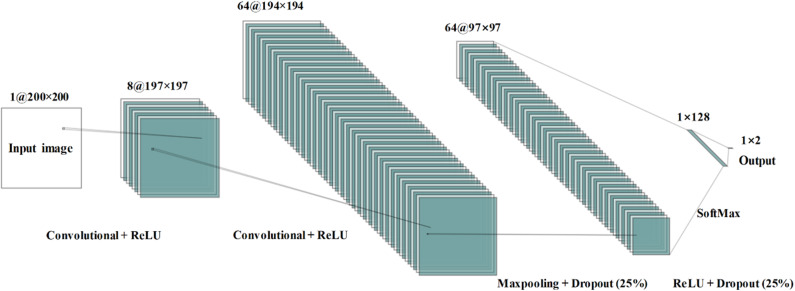
Convolutional neural network architecture used during the study for mild cognitive impairment and healthy control classification.

The architecture of the proposed CNN model contains four layers that two convolutional layers and two fully connected layers—as shown in Figure 6. In order for the input size to be consistent, the input neural image size is set to 200 × 200. The number of kernels is eight; the kernel size is 4 × 4; the size of the pooling area is 2 × 2; the value of the stride is 1. There were 128 neurons in the first fully connected layer with the activation function of *ReLu*. The loss function employed is categorical cross-entropy. Adam optimization was used to select the adaptive learning rate and the parameters during gradient descent.

In this study, the CNN model may have suffered from an overfitting problem because of the limitation of the sample data. We employed 5-fold cross-validation to decrease the influence of this problem on the experiment results. The data were randomly divided into 5-folds. One subsample fold was selected to test the performance of the trained model, and the remaining 4-folds were set to train the CNN model. This process was reiterated five times to ensure that each subsample was utilized as a validation set once.

## Results

### Hemodynamic Response and Behavioral Result

As shown in Table 1, the statistical analysis of the behavioral measurement (i.e., the averaged K-MMSE score) was performed by two independent sample *t*-test with a significant level of 0.05. The result (*p* = 0.49) presents a negative correlation between the behavioral state and the real subject mental state. In this study, we analyzed 3,456 fNIRS channels (i.e., 24 subjects × 3 trials × 48 channels) for each task. As shown in Table 2, the total number of selected ROI channels (activated channels) was 1,826 (*N*-back task), 1,609 (Stroop task), and 1,867 (VFT). The percentage of activation/deactivation was calculated by dividing the number of the ROI channels by the total number of channels. Therefore, the percentages of activated patterns are 52.83% (*N*-back task), 46.56% (Stroop task), and 54.02% (VFT), respectively.

**Table 2:** Number of ROI channels of each subject for three mental tasks (i.e., *N*-back task, Stroop task, and verbal fluency task).

**Subject**	***N*****-back task**			**Stroop task**			**Verbal fluency task**		
	**Trial 1**	**Trial 2**	**Trial 3**	**Trial 1**	**Trial 2**	**Trial 3**	**Trial 1**	**Trial 2**	**Trial 3**
1	17	23	29	31	33	36	39	27	37
2	32	26	19	23	19	15	28	32	32
3	36	33	39	32	34	35	19	32	31
4	23	31	18	12	12	18	6	23	20
5	10	37	32	20	9	8	11	16	18
6	21	22	16	12	25	22	27	34	24
7	16	30	21	13	38	23	40	37	27
8	15	15	25	9	17	16	19	31	29
9	27	27	20	21	33	22	18	11	17
10	35	10	30	38	7	24	40	41	24
11	40	36	35	39	33	38	28	35	39
12	31	28	28	19	30	21	11	22	8
13	44	31	35	37	35	24	3	22	34
14	26	22	28	38	10	6	28	22	31
15	35	1	30	36	25	13	3	17	30
16	23	8	11	19	30	21	25	17	25
17	8	16	9	20	14	15	22	29	27
18	47	26	41	8	17	20	42	39	40
19	28	40	27	41	15	14	21	19	15
20	23	14	1	31	30	30	1	27	46
21	16	28	38	20	3	3	33	28	28
22	40	2	2	6	10	12	10	30	31
23	30	43	21	13	30	18	20	24	31
24	21	31	47	36	48	24	36	35	43
Total	1,826			1,609			1,867		

**Figure 7:**
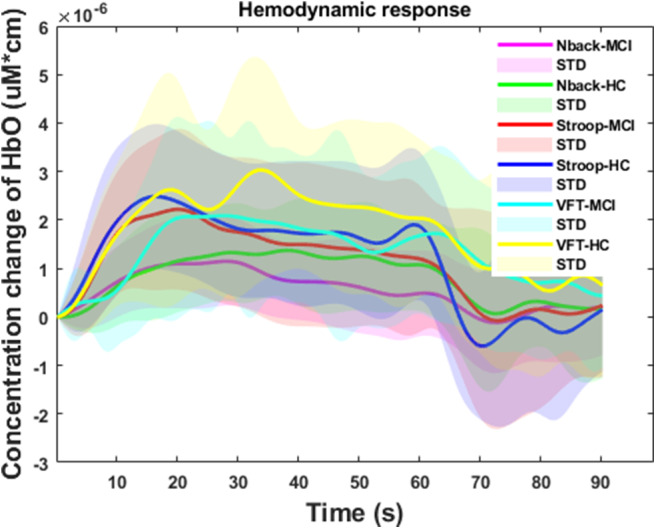
Temporal feature of the ΔHbO for three mental tasks (i.e., *N*-back task, Stroop task, and verbal fluency task) for mild cognitive impairment and healthy control groups, respectively.

Figure 7 summarizes the averages and standard deviations (STDs) of the hemodynamic response from the ROI channels of the patients with MCI and the HCs during various mental tasks—*N*-back task, Stroop task, and VFT. The solid lines refer to the mean of the ΔHbO, and shaded areas represent the STD of ΔHbO among the subjects. To compare the unique patterns (i.e., an increase or a decrease) of the hemodynamic response of the HCs and the MCI individuals in the *N*-back task (MCI: solid magenta line and HC: solid green line), Stroop task (MCI: solid red line and HC: solid blue line), and VFT task (MCI: solid cyan line and HC: solid yellow line), respectively, we applied two independently sampled *t*-tests. The results indicate that the average hemodynamic response of MCI patients is significantly lower than that of HCs in the *N*-back task (*p* < 0.001) and VFT task (*p* < 0.001). In the Stroop task, the average hemodynamic change in MCI individuals appears to be similar to that of HCs (*p* = 0.06825).

### Neural Images in Spatial and Temporal-Spatial Domain

**Figure 8:**
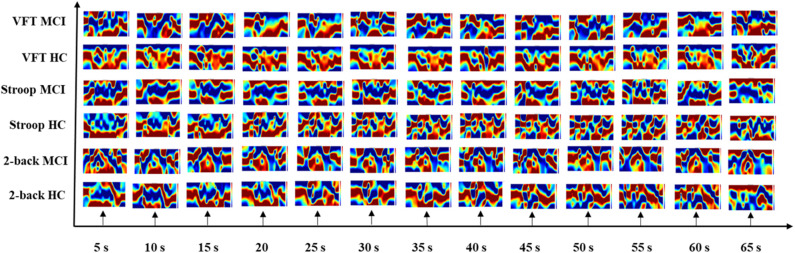
Neuroimaging of specific time points (i.e., 5, 10, 15, 20, 25, 30, 35, 40, 45, 50, 55, 60, and 65 s) in spatial domain among three mental tasks (i.e., *N*-back task, Stroop task, and verbal fluency task).

**Figure 9:**
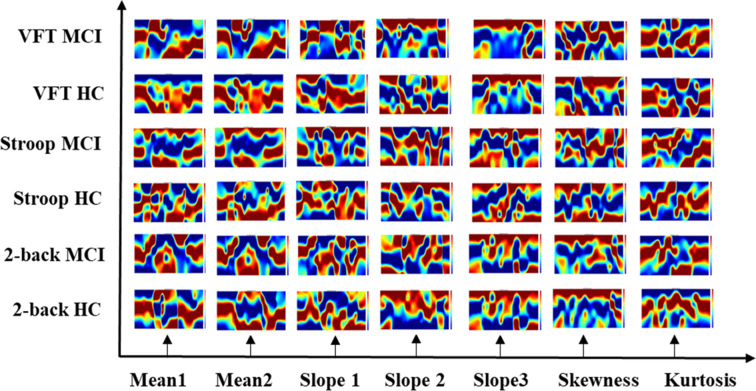
Neuroimaging of temporal features (i.e., mean value from 5 to 65 s, mean value from 5 to 25 s, slope value from 5 to 15 s, slope value from 20 to 60 s, slope value from 60 to 70 s, skewness value from 5 to 65 s, and kurtosis from 5 to 65 s) in spatial domain.

The results of the neuroimaging that was conducted based on the concentration change in oxygen-hemoglobin of the specific time point (i.e., 5, 10, 15, 20, 25, 30, 35, 40, 45, 50, 55, 60, and 65 s), are presented in Figure 8. The neural activation slightly changed over time in the entire prefrontal cortex. It is also easily observed that the MCI group displays a lower neural activation than the HCs in the three mental tasks. Figure 9 illustrates the neuroimaging created by the temporal features (i.e., mean values from 5 to 65 s and from 5 to 25 s, slopes from 5 to 15 s, from 20 to 60 s, and from 60 to 70 s, skewness from 5 to 65 s, and kurtosis from 5 to 65 s) in the spatial domain. As compared to Figure 8, the patterns of the neural images of temporal features in the spatial domain area differ; for example, the neural images in Figure 8 are highly correlated, and the neural images generated by each temporal feature in Figure 9 have their individual characteristics. Interestingly, the neural images generated by the mean values also display the same patterns as characteristics generated by the specific time points. Among the three mental tasks, the VFT task shows the highest neural activation pattern in the HC group. The lowest neural activation is shown by the Stroop task in the MCI group. In addition, the neural images at 5 s among the six groups show lower neural firing than the neural images at 10 s and at other time points. In contrast, neural images at 60 s and previous time points show higher neural firing than those at 65 s.

### CNN Results for Classification of Neural Images

The input dataset of the temporal feature contained 24 (subjects) × 3 (trials) × ROI channels. In the neural images in the spatial and temporal-spatial domain cases, each category had a dataset of size 24 (subjects) × 3 (trials). To verify the CNN's capability to classify MCI individuals and HCs, we utilized the standard metrics (accuracy, recall, precision, and F1-score) (Powers, 2011; Lin et al., 2018) to assess the results. Their definitions are

(9) $$\begin{array}{l} \text{Accuracy}=\frac{\text{TP}+\text{TN}}{\text{TP}+\text{TN}+\text{FP}+\text{FN}}, \end{array}$$

(10) $$\begin{array}{l} \text{Recall}=\frac{\text{TP}}{\text{TP}+\text{FN}}, \end{array}$$

(11) $$\begin{array}{l} \text{Precision}=\frac{\text{TP}}{\text{TP}+\text{FP}}, \end{array}$$

(12) $$\begin{array}{l} \text{F}1\text{score}=2\times \;\frac{\text{Precision}\times \text{Recall}}{\text{Precision}+\text{Recall}}\;, \end{array}$$

where TP, TN, FP, and FN represent the true positive, true negative, false positive, and false negative, respectively. In this study, TP indicated the number of MCI patients correctly classified; TN is the number of HCs identified correctly; FP refers to the number misclassified as MCI patients, and FN is the number misclassified as HCs.

The CNN results for the dataset extracted by the temporal domain are listed in Table 3. The average accuracy among the three mental tasks is 80.15% with an STD of 3.95%, and the results of recall, precision, and F1-score are 73.26% (4.10%), 64.23% (6.63%), and 67.48% (5.40%), respectively. Stroop achieved a higher accuracy rate than both the *N*-back task and VTF: 84.70%.

**Table 3:** Convolutional neural network results of the temporal features among three mental tasks (i.e., *N*-back task, Stroop task, and verbal fluency task).

**Task**	**Accuracy (%)**	**Recall (%)**	**Precision (%)**	**F1-score (%)**
*N*-back task	77.90	70.00	58.95	63.22
Stroop task	84.70	77.87	71.67	73.55
VFT	77.84	71.91	62.07	65.66
Average	80.15	73.26	64.23	67.48
STD	3.95	4.10	6.63	5.40

Tables 4–6 depicts the CNN's performance for neural images at various time points in the spatial domain of the *N*-back task, Stroop task, and VFT, respectively. CNN's classification performance is divided into four different categories, as mentioned previously: accuracy (%), recall (%), precision (%), and F1-score (%). Furthermore, for each category, the results for specific time points are shown in the first column. Table 4 represents CNN classification results of the *N*-back task. For the *N*-back task, the accuracy rate ranges from 65.28 to 93.06%, recall from 55.66 to 60%, precision from 41.11 to 86.43%, and F1-score from 46.34 to 87.83%. The classification result at the 15 s time point shows the best performance. The average accuracy rate is 82.59%. In the Stroop task case (shown in Table 5), the best performance appeared at the 60 s time point. Interestingly, this is consistent with the results for the hemodynamic response in Figure 7, i.e., there is a peak at the 60 s time point in the case of the Stroop task. The lower accuracies occur at 5 s (76.86%), 20 s (75.43%), 35 s (78.57%), and 65 s (77.71%). The average accuracy is 85.03%. In contrast, at the 60 s time point, the worst results (65.28%) were obtained in the Stroop task. The higher accuracies appear at the time points of 5 s (91.43%), 15 s (92.00%), and 50 s (92.00%) during the VFT task (shown in Table 6). These results are in accordance with the hemodynamic response in Figure 7 (solid yellow line and cyan solid line). The average accuracy is 82.20%. Among the results of the three mental tasks, the four verifying factors are always congruent. This means that when the accuracy rate is higher, the values of the corresponding recall, precision, and F1-score are also higher. For instance, when the highest accuracy is 98.57%, the highest recall (98.89%), precision (98.33%), and F1-score (98.50%) also appear for the same features.

**Table 4:** Convolutional neural network classification results of neuroimaging with spatial features for the *N*-back task.

**Features**	**Accuracy (%)**	**Recall (%)**	**Precision (%)**	**F1-score (%)**
HbO map at 5 s	77.78	70.00	58.86	63.15
HbO map at 10 s	91.67	90.00	86.00	87.50
HbO map at 15 s	93.06	90.00	86.43	87.83
HbO map at 20 s	84.72	80.00	72.43	75.33
HbO map at 25 s	76.39	70.00	58.43	62.83
HbO map at 30 s	77.78	70.00	58.43	62.83
HbO map at 35 s	77.78	70.00	58.43	62.83
HbO map at 40 s	91.67	90.00	86.00	87.50
HbO map at 45 s	77.78	70.00	58.43	62.83
HbO map at 50 s	93.06	90.00	86.43	87.83
HbO map at 55 s	84.72	80.00	72.43	75.33
HbO map at 60 s	65.28	55.66	41.11	46.34
HbO map at 65 s	81.94	78.00	71.00	73.36
Average	82.59	77.20	68.80	71.96

**Table 5:** Convolutional neural network classification results of neuroimaging with spatial features for the Stroop task.

**Features**	**Accuracy (%)**	**Recall (%)**	**Precision (%)**	**F1-score (%)**
HbO map at 5 s	76.86	70.00	58.43	62.83
HbO map at 10 s	90.57	88.89	84.33	86.00
HbO map at 15 s	83.43	78.89	70.76	73.83
HbO map at 20 s	75.43	68.89	56.76	61.33
HbO map at 25 s	84.86	80.00	72.43	75.33
HbO map at 30 s	92.00	90.00	86.00	87.50
HbO map at 35 s	78.57	70.00	59.28	63.48
HbO map at 40 s	84.86	80.00	72.43	75.33
HbO map at 45 s	84.00	80.00	72.00	75.00
HbO map at 50 s	85.71	80.00	72.86	75.65
HbO map at 55 s	92.86	90.00	86.43	87.83
HbO map at 60 s	98.57	98.89	98.33	98.50
HbO map at 65 s	77.71	70.00	58.86	63.15
Average	85.03	80.43	72.99	75.83

**Table 6:** Convolutional neural network classification results of neuroimaging with spatial features for the VFT task.

**Features**	**Accuracy (%)**	**Recall (%)**	**Precision (%)**	**F1-score (%)**
HbO map at 5 s	91.43	88.89	84.76	86.33
HbO map at 10 s	77.71	70.00	58.86	63.15
HbO map at 15 s	92.00	90.00	86.00	87.50
HbO map at 20 s	70.57	60.00	45.29	50.98
HbO map at 25 s	77.71	70.00	58.86	63.15
HbO map at 30 s	84.86	80.00	72.43	75.33
HbO map at 35 s	77.71	70.00	58.86	63.15
HbO map at 40 s	78.57	70.00	59.29	63.48
HbO map at 45 s	84.86	80.00	72.43	75.33
HbO map at 50 s	92.00	90.00	86.00	87.50
HbO map at 55 s	78.57	70.00	59.29	63.48
HbO map at 60 s	84.86	80.00	72.43	75.33
HbO map at 65 s	77.71	70.00	58.86	63.15
Average	82.20	76.07	67.18	70.60

The CNN classification results of neuroimaging of temporal features in the spatial domain are shown in Tables 7–9. The first column of “mean map (5:65 s),” “mean map (5:25 s),” “slope map (5:15 s),” “slope map (20:60 s),” “slope map (60:70 s),” “kurtosis map (5:65 s),” and “skewness map (5:65 s)” represents the neural map generated based on a mean value of 5–65 s, mean value of 5–25 s, slope value of 5–15 s, slope value of 20–60 s, slope value of 60–70 s, kurtosis of 5–65 s, and skewness of 5–65 s, respectively. In comparison, in the results of neural imaging of spatial features, the average accuracies are higher for all three mental tasks (i.e., *N*-back task: 89.46%, Stroop task: 88.00%, and VFT: 90.37%) as shown in Tables 7–9, respectively. In the *N-*back task, the highest accuracy (98.61%) occurred in the slope map from 20 to 60 s, and the lowest accuracy appeared in the mean map (5–25 s). The highest accuracy is 98.57% in the slope map (5–15 s) during the Stroop task, and the lowest accuracy is 77.71% in the slope map (20–60 s). For the VFT task, the accuracy range is from 84.86% (mean map during 5–65 s and skewness map) to 98.57% (mean map 5–25 s).

**Table 7:** Convolutional neural network classification results of neuroimaging with temporal-spatial features for the *N*-back task.

**Features**	**Accuracy (%)**	**Recall (%)**	**Precision (%)**	**F1-score (%)**
Mean map (5:65 s)	84.72	80.00	72.43	75.33
Mean map (5:25 s)	76.39	70.00	58.43	62.83
Slope map (5:15 s)	91.67	90.00	86.00	87.50
Slope map (20:60 s)	98.61	98.89	98.33	98.50
Slope map (60:70 s)	92.86	90.00	86.42	87.83
Skewness map (5:65 s)	91.67	90.00	86.00	87.50
Kurtosis map (5:65 s)	90.28	88.89	84.33	86.00
Average	89.46	86.83	81.71	83.64

**Table 8:** Convolutional neural network classification results of neuroimaging with temporal-spatial features for the Stroop task.

**Features**	**Accuracy (%)**	**Recall (%)**	**Precision (%)**	**F1-score (%)**
Mean map (5:65 s)	92.86	90.00	86.43	87.83
Mean map (5:25 s)	91.43	88.89	84.76	86.33
Slope map (5:15 s)	98.57	98.89	98.33	98.50
Slope map (20:60 s)	77.71	70.00	58.86	63.15
Slope map (60:70 s)	85.71	80.00	72.86	75.65
Skewness map (5:65 s)	84.86	80.00	72.43	75.33
Kurtosis map (5:65 s)	83.43	78.00	71.43	73.69
Average	87.80	83.68	77.87	80.07

**Table 9:** Convolutional neural network classification results of neuroimaging with temporal-spatial features for the VFT task.

**Features**	**Accuracy (%)**	**Recall (%)**	**Precision (%)**	**F1-score (%)**
Mean map (5:65 s)	84.86	80.00	72.43	75.33
Mean map (5:25 s)	98.57	98.89	98.33	98.50
Slope map (5:15 s)	92.86	90.00	86.43	87.83
Slope map (20:60 s)	85.71	80.00	72.86	75.65
Slope map (60:70 s)	92.86	90.00	86.43	87.83
Skewness map (5:65 s)	92.86	90.00	86.43	87.83
Kurtosis map (5:65 s)	84.86	80.00	72.43	75.33
Average	90.37	86.98	82.19	84.04

## Discussion

The objective of the study was to investigate the neuroimaging biomarkers and select the possible candidate biomarkers for the early detection of AD. To attain this goal, we examined neural images that were generated based on 3 temporal features, 13 spatial features, and 7 temporal-spatial features for training the CNN model, respectively. Finally, we suggest the use of two temporal-spatial features (mean map, slope map) for identification of MCI patients due to the high classification accuracy (90.37%, the averaged accuracy of VFT, Table 9). Especially, the slope map from 20 to 60 s with the *N*-back task achieved the highest accuracy of 98.61%, see Table 7, Slope map (20:60 s). It is the first study to assess neural images obtained by fNIRS signals for early AD detection. Furthermore, the results obtained for MCI detection constitute the highest diagnosis performance in fNIRS areas. Interpretable, non-invasive, reliable, low cost, and portable biomarkers are always the necessary tools for the identification of patients with MCI symptoms. The computer-aided neural imaging method could provide a novel direction for the clinical diagnosis of MCI.

fNIRS, a novel non-invasive neuroimaging modality, has proven its worth during the last decade, especially in the healthcare industries (Khan et al., 2018; Hong and Yaqub, 2019). The first article on the use of fNIRS for MCI detection appeared in 2006. This paper proposed that MCI patients show a decreased ΔHbO in the right parietal cortex during the VFT (Arai et al., 2006), and it was the first study to suggest fNIRS as a potential tool for screening AD/MCI. After 7 years, more related papers were published. One of the articles proved that the difference in ΔHbO of MCI individuals and HCs could also be measured in the prefrontal cortex (Doi et al., 2013). In addition, the ΔHbO during the resting state (Viola et al., 2013) and *N*-back task (Niu et al., 2013) also presents signs of neurodegeneration in the MCI group. In 2014, abnormal metabolisms of MCI were observed by some clinical research groups (Babiloni et al., 2014; Liu et al., 2014), such as the hypercapnia effect, global brain hypoperfusion, oxygen hypometabolism, and neurovascular decoupling. Later, the verification of neurodegeneration was also extended to the other cerebral brain regions, i.e., prefrontal cortex (Uemura et al., 2016; Yeung et al., 2016b; Vermeij et al., 2017) inferior frontotemporal cortex (Katzorke et al., 2018), and lateral prefrontal cortex (Marmarelis et al., 2017). Meanwhile, some researchers (Yeung et al., 2016a; Yap et al., 2017; Li et al., 2018a,b,c; Zeller et al., 2019) started to explore reliable biomarkers, i.e., complexity, number of activated channels, mean value of ΔHbO, time to reach peak value, and slope, and lateralization hyperactivation patterns. Interestingly, neurodegeneration symptoms similar to those mentioned above could be repeated. By virtue of these studies, novel directions for understanding the neurological information of MCI symptoms better were provided. Moreover, the relative results also proved that fNIRS is a promising tool for detecting the difference between MCI individuals and HCs. In this study, the hemodynamic responses (shown in Figure 7) of ΔHbO during three tasks, the *N*-back task, the Stroop task, and VFT, were consistent with the result of the researches (Yap et al., 2017; Li et al., 2018b) related to AD/MCI detection. Interestingly, the percentages of the activated channels (i.e., *N*-back: 52.83%, Stroop: 46.56%, and VFT: 54.02%) were similar to Mandrick et al. (2013), in which the percentage of activation in the prefrontal cortex during the mental and motor tasks was 51%. However, the provision of a diagnostic decision based on the group difference presents a challenge, because a high STD exists between the two groups.

As mentioned above, researchers prefer to observe the hemodynamic response in time series. Typically, the fluctuation of concentration changes in oxygenated hemoglobin provides evidence for differences in metabolism between the two groups. However, this technique suffers from at least two limitations. (1) Poor robustness: Because of the disturbance of the noise and some intrinsic physiological causes, there is a high possibility that some channels will not be activated or fully activated by noise (Birn, 2012; Wald and Polimeni, 2017). To a large extent, the affected channel would influence the fluctuation of ΔHbO/ΔHbR. (2) Loss of information from the brain network: According to the recent literature (Fornito and Harrison, 2012), the neural disorder is associated with subtle abnormalities distributed throughout the brain. Studies have implied that the neurodegeneration arises from disordered interaction in the connected neural system rather than in the focal channel (Breakspear and Jirsa, 2007). Thus, neglecting the spatial (network level) domain would lead to a significant loss in terms of understanding better the symptom of neural disorders. This comparison was also evaluated in our previous publication (Yang et al., 2019). As our initial investigated indicated, the digital biomarkers, which were obtained based on ROI channels, showed lower accuracy than the image biomarkers (network level). Similarly, the difference of the hemodynamic response for the MCI and HC groups is easy to observe in the spatial domain, which is demonstrated in Figures 8, 9.

To meet the demand for clinical diagnoses, it is crucial to converting the neural images into a form that allows an interpretable clinical decision (Martinez-Murcia et al., 2018). With the development of machine/deep learning methods, researchers have started to utilize machine/deep learning for identifying AD/MCI, because the natural images and brain images have similarities (Vieira et al., 2017). In the recent literature (Ieracitano et al., 2018; Ju et al., 2019), it was claimed that deep learning methods (e.g., CNN) would present a superiority for diagnosis for AD/MCI by using EEG and fMRI signals. Also, in our initial fNIRS study (Yang et al., 2019), we compared the statistical analysis, LDA, and CNN for identifying MCI patients from HCs. The results are consistent with the EEG (Ieracitano et al., 2018) and fMRI studies (Ju et al., 2019) that deep learning methods have a better performance than LDA/statistical analysis. Therefore, in this study, we employed the CNN and evaluated the temporal, spatial, temporal-spatial biomarkers for MCI diagnosis.

One of the first studies (Gupta et al., 2013) in which a CNN was applied to structural MRI data achieved classification accuracy rates of 94.7% for AD vs. HC and 86.4% for MCI vs. HC. Thus far, with the development of DL, the classification accuracy has reached a high accuracy for MRI (98/99%) (Khagi et al., 2019), EEG (98.4%) (Amezquita-Sanchez et al., 2016), and fMRI (97%) (Hojjati et al., 2018). According to the results shown in Tables 4–9, the average classification accuracy (i.e., *N*-back task: 89.46%, Stroop task: 88.00%, and VFT: 90.37%) in the temporal and spatial domain could prove that fNIRS is also a promising diagnostical modality. In comparison to our previous study, which utilized the image biomarkers (*t*-map and connectivity map) for MCI identification with fNIRS signals (Yang et al., 2019). Our current classification accuracy (highest accuracy: 98.61%) is further improved, and more reliable image biomarkers (e.g., mean map and slope map) are provided for the clinical diagnosis.

Among the three mental tasks, there was no significant difference based on the CNN classification performance. In comparison with the results of the mental task, the feature selection seems considerably more informative. The performance yielded by the temporal-spatial feature is superior to that yielded by the temporal and spatial features. According to our results, the temporal feature (i.e., the slope map between 5 and 15 s) always showed a good performance. The possible reason is the lower hemodynamic response of people with MCI during the initial stage of the task, which in turn explains why the slope value of the HC group increases faster than that of the MCI group. To reach a more reliable and precise decision, we suggest utilizing a combination of features.

Although the present study has proposed and evaluated the imaging biomarkers for MCI detection with the fNIRS signals using the CNN method (i.e., the highest accuracy was 98.61%), some limitations need be mentioned. First, the fNIRS signals were measured only from the prefrontal cortex, since the benefit of no hair in the prefrontal region can minimize the scattering and attenuation effects. A different result with different biomarkers might also be observed from other brain regions (e.g., the parietal cortex). Derosière et al. (2014) have shown that the parietal cortex revealed a better classification accuracy than the prefrontal cortex for attention state classification. Therefore, a combination of both prefrontal and parietal cortices will provide an improved classification accuracy for MCI case too. Second, the exact location of the FpZ reference point in the International 10–20 System might not have been observed consistently, because the fNIRS device (NIRSIT, OBELAB Inc., Republic of Korea) had fixed emitter-detector distances, and the head shapes of individual subjects were not the same. In addition, data augmentation might be another way to avoid CNN overfitting issues. In this study, we used two drop out layers to overcome the overfitting problem, and the loss plot showed that the overfitting did not appear. The data augmentation method will be considered in our future work to deal with the overfitting issue. For the investigation of the spatial feature, we randomly selected 13 specific time points as features for conducting the comparison. Because of the limitation of the computation, it is difficult to list all the time points (i.e., 8.138 Hz × 90 s = 732 points) and conduct the process as mentioned above. This limitation could be compensated by using a real-time analysis system or dividing more time segments by a shorter time window (i.e., 5–15 s). Likewise, in the temporal-spatial features, the time point selection also presents a challenge. In general, a neural image based on the first 15 s always yields a good accuracy level. Driving more time points in the time windows between 5 and 15 s would like to be done in future work to reduce the processing time. In the real clinical application, the biggest challenge is data training: However, once the model gets appropriately trained, the system can be used as a reliable tool for diagnosing MCI patients.

## Conclusion

In this study, we highlighted the feasibility of using fNIRS for the early detection of AD by using neural imaging based on the temporal, spatial, and temporal-spatial features. This systematically analyzed results indicate that neural imaging of the combined temporal and spatial features (i.e., the average accuracy of *N*-back: 89.46%, Stroop: 87.80%, and VFT: 90.37%) produces a more reliable performance than those when using temporal and spatial features separately. In particular, the slope map (20–60 s) during the *N*-back task achieved the highest accuracy of 98.61%. In the Stroop task and VFT case, the maximum accuracy is 98.57% by the slope map (5–15 s) and mean map (5–25 s). Besides that, all the mental tasks could achieve a good accuracy (>90%) within the time windows (5–15 s). This finding provides the possibility to use the short time windows for early detection of the AD. Conclusively, our results indicate that the CNN-aided temporal-spatial neuroimaging method could assist the clinical diagnosis of MCI. Additionally, the classification performance based on the spatial neural image also provides a possibility of reducing the diagnosis time in future studies.

## Data Availability Statement

The datasets generated for this study are available on request to the corresponding author.

## Ethics Statement

The studies involving human participants were reviewed and approved by Pusan National University Institutional Review Board. The patients/participants provided their written informed consent to participate in this study.

## Author Contributions

DY conducted the data analysis and wrote the first draft of the manuscript. RH and S-HY participated in the initial data analysis. M-JS and JY interviewed the participants and managed the processes related to experimentation and interventions. Y-IS designed the initial experimental paradigm. K-SH suggested the theoretical aspects, corrected the manuscript, and supervised all the process from the beginning. All authors have approved the final manuscript.

## Conflict of Interest

The authors declare that the research was conducted in the absence of any commercial or financial relationships that could be construed as a potential conflict of interest.
